# One-Year Mortality After Percutaneous Endoscopic Gastrostomy: The Prognostic Role of Nutritional Biomarkers and Care Settings

**DOI:** 10.3390/nu17050904

**Published:** 2025-03-05

**Authors:** Nermin Mutlu Bilgiç, Güldan Kahveci, Ekmel Burak Özşenel, Sema Basat

**Affiliations:** 1 Department of Gastroenterology, University of Health Sciences, Ümraniye Training and Research Hospital, 34764 Istanbul, Türkiye; 2 Department of Nutritional Nursing, University of Health Sciences, Ümraniye Training and Research Hospital, 34764 Istanbul, Türkiye; 3 Department of Internal Medicine, University of Health Sciences, Ümraniye Training and Research Hospital, 34764 Istanbul, Türkiye

**Keywords:** care settings, enteral nutrition, inflammation, mortality, nutritional status, percutaneous endoscopic gastrostomy, prognostic nutritional index

## Abstract

**Background/Objectives**: This study aimed to evaluate the clinical outcomes, complications, and one-year mortality of patients undergoing percutaneous endoscopic gastrostomy (PEG) in different care settings (hospital, nursing home, and home). Additionally, we investigated the comparative prognostic role of the prognostic nutritional index (PNI) and the CRP-to-albumin ratio (CAR) in predicting mortality among these patients. **Methods**: A retrospective analysis of 236 adult patients who underwent PEG placement between January 2022 and December 2023 was performed. Demographic, clinical, and laboratory data were collected. The PNI was calculated according to the following formula: PNI = 10 × (albumin) + 0.005 × (lymphocyte count). The CAR was obtained by the ratio of the CRP level to the albumin level. Patients were categorized based on their post-PEG care settings. **Results**: Neurologic disorders were the most common indication for PEG (69.9%). The one-year mortality was 32.2%, with a median survival of 38 weeks (95% CI: 35–41). In the multivariable model, a lower PNI (HR = 0.93, 95% CI: 0.89–0.97, *p* < 0.001), as well as being followed in a hospital setting, emerged as independent predictors of mortality. Patients with timely PEG tube replacement showed a reduced mortality risk. The ROC analysis showed that the PNI had a higher AUROC (0.78 ± 0.04) compared to the CAR (0.69 ± 0.04), indicating superior prognostic accuracy for predicting one-year mortality. **Conclusions**: Care settings significantly influence survival outcomes, with better mortality rates observed in nursing homes and home environments. The PNI was superior to the CAR in predicting one-year mortality, emphasizing its clinical utility in risk stratification for PEG patients. Proactive tube management and individualized care strategies are critical for improving the prognosis in this population.

## 1. Introduction

Percutaneous endoscopic gastrostomy (PEG) is a minimally invasive procedure designed to enable long-term enteral feeding in patients with difficulties swallowing caused by conditions such as neurological disorders, stroke, dementia, motor neuron diseases, head injuries, or malignancies of the oropharynx and esophagus, as well as those unable to meet nutritional needs through the gastrointestinal tract [1]. First described by Gauderer et al. in 1980, PEG is now widely performed safely without requiring surgical intervention [2]. While PEG placement is considered a generally safe procedure, it may result in minor complications such as skin irritation, granulation tissue formation, pneumoperitoneum, tube obstruction or displacement, and wound infections [3]. Major complications include buried bumper syndrome, infections causing peritonitis or necrotizing fasciitis, bleeding, and injury to internal organs [4].

After PEG placement, the clinical outcomes of patients followed in hospital or home settings, as well as the knowledge levels and practices of caregivers regarding enteral nutrition, tube care, and complications, are of critical importance [5,6]. Inadequate care at home can heighten the risk of minor and major complications, while prolonged hospital stays can lead to hospital-acquired infections and increased costs [7]. Additionally, a prolonged PEG tube duration can also increase the risk of complications [8]. Despite these challenges, comparative studies on the prognosis of PEG patients in hospital versus home settings remain scarce in the literature. Recent evidence suggests that patients monitored in hospital settings tend to have higher mortality rates than those followed at home or in nursing homes [9,10,11]. This disparity may stem from the severity of underlying conditions, differences in nutritional and inflammatory status, and increased exposure to nosocomial complications in hospital settings. Additionally, better-trained caregivers and structured enteral nutrition management in non-hospital settings may contribute to improved long-term outcomes [12,13].

The prognostic nutritional index (PNI), calculated based on albumin levels and the lymphocyte count, serves as an effective indicator of nutritional and immunological status linked to prognosis in various diseases [14,15,16,17,18]. In recent studies involving patients who had undergone PEG, the PNI has been found to be associated with early mortality [19,20]. The prognostic role of the PNI for patients who had undergone PEG and who were followed up in various settings, such as homes, nursing homes, and hospitals, has yet to be compared.

We hypothesized that variations in the sociodemographic characteristics of caregivers, such as the education level of patients who had undergone PEG and who were monitored in different settings, might impact malnutrition, complications, and mortality rates. The primary aim of this study was to evaluate the complications related to PEG placement and the one-year survival rate among adult patients who underwent this procedure. The secondary aim was to assess the prognostic role of the PNI in predicting mortality in these patients across various care environments (homes, nursing homes, and hospitals).

## 2. Materials and Methods

This retrospective study was conducted on patients who had undergone PEG in the Gastroenterology Clinic of Ümraniye Education and Research Hospital, University of Health Sciences, between January 2022 and December 2023. This study adhered to the ethical regulations and principles specified in the Declaration of Helsinki and received approval from the Ethical Committee of Ümraniye Education and Research Hospital (Date: 8 February 2024, Decision No: 58). The requirement for obtaining informed consent was exempted by the Ethics Committee due to the retrospective design of the study.

### 2.1. Study Population

Due to the retrospective nature of the study, a traditional prospective sample size calculation was not performed prior to data collection. Instead, to maximize the study’s power and representativeness, all patients who underwent PEG between January 2022 and December 2023 were evaluated for eligibility. A total of 258 patients who underwent PEG were retrospectively evaluated for study eligibility. Indications for PEG were identified based on standard guidelines and included cancer, cerebrovascular event (stroke), Parkinson’s disease, Alzheimer’s disease, dementia, femur fracture, hemorrhage, hydrocephalus, hypoxic encephalopathy, cerebral palsy, and trauma-related conditions. The exclusion criteria included patients under 18 years of age (n = 2), those for whom PEG placement was technically unfeasible (n = 5), those with contraindications to PEG (n = 3), those with a poor prognosis (life expectancy under one month) (n = 4), and those with missing data (n = 8). After applying the exclusion criteria, 236 patients were included in the final analysis.

### 2.2. Data Collection

Patient data were collected using the hospital’s electronic information system and patient files. Demographic, clinical, and outcome parameters were retrieved and recorded in a standardized electronic spreadsheet. All collected variables were fully available for the included patients, ensuring no missing data in the analyzed dataset. Collected variables included demographic futures (age and gender), PEG-related data (date of the PEG placement, number of PEG changes, time intervals between changes, and an indication for PEG), the care environment (home, nursing home, or hospital care), caregiver characteristics (age, gender, level of education, and number of caregivers involved), nutritional management (product and type), PEG site care, complications related to PEG placement and follow-up (leakage around the stoma site, tube dislodgement, tube deformation, occlusion, and tearing), laboratory parameters, one-year follow-up survival status and, if applicable, time to death.

### 2.3. Laboratory Measurements

Venous blood samples were collected from all patients before the PEG procedure, following an 8 h fasting period. These samples were then analyzed using the Mindray MC6800 (Mindray, Shenzhen, China) and Architect Plus (Abbott Diagnostics, Abbott Park, IL, USA) devices. Levels of leukocytes (the impedance method), *C*-reactive protein (CRP) (the immunoturbidimetric method), and albumin (the bromocresol green method) were determined. PNI was calculated according to the following formula: PNI = 10 × (albumin level in g/dL) + 0.005 × (total lymphocyte count) [21]. Additionally, the CAR was obtained by the ratio of the CRP level to the albumin level.

### 2.4. PEG Procedure

All PEG procedures were performed using the pull (traction) method under the supervision of an anesthesiologist in our endoscopy unit after an 8 h fasting period with continuous monitoring. Prior to the procedure, all patients received local anesthesia (prilocaine hydrochloride) and sedation (midazolam 0.05 mg/kg). Prophylactic antibiotics were administered to all patients. To protect the transverse colon, an enema was performed in immobilized patients with significant bowel distension before the procedure. The gastroduodenoscopy was performed using a fiber endoscope, and the upper gastrointestinal system was examined up to the second part of the duodenum to rule out any pathology that might obstruct PEG placement. The area designated for catheter insertion was disinfected with povidone-iodine and locally anesthetized. Using transillumination, an appropriate insertion site on the abdominal wall was identified. The second operator confirmed the location by pressing on the selected site with a finger while it was visualized endoscopically. A needle was then introduced into the stomach, followed by the insertion of a guidewire, which was grasped with a snare and pulled out through the mouth, allowing the gastrostomy tube to be guided into the stomach. An 18–20 Fr PEG set was used for the procedure. The PEG tube was secured in place, ensuring it could rotate freely within the abdominal wall. A final endoscopic examination was performed to verify proper positioning, followed by hemostasis control before concluding the procedure.

### 2.5. Follow-Up Protocol

For the hospital group, patients remained in the hospital if they required complex medical management, frequent monitoring, or were unable to safely transition to home or nursing home care. For the nursing home group, patients in institutional care were managed by on-site medical and nursing teams and were routinely monitored by either a gastroenterologist or a nutrition specialist. For the home group, patients were discharged to their residences and followed by outpatient visits or home care teams. Caregivers received detailed training on enteral feeding administration, tube care, and complication recognition. PEG replacement was conducted in accordance with the ESPEN guidelines, and the relevant protocols were followed [22].

### 2.6. Outcome Measures

The primary outcome of this study was to evaluate the complications related to PEG placement and the one-year survival rate following the procedure. The secondary outcome was to determine the prognostic value of the PNI in predicting mortality in patients receiving care in different settings, including homes, nursing homes, and hospital environments.

### 2.7. Statistical Analysis

All data were analyzed using IBM SPSS Statistics for Windows, version 20.0 (IBM Corp., Armonk, NY, USA). Numerical data that were determined to be normally distributed based on the results of the Kolmogorov–Smirnov tests are presented as mean ± standard deviation (SD) values. In contrast, non-normally distributed variables are presented as median (min-max) values. ANOVA or Kruskal–Wallis tests were used to compare continuous variables among three care settings (homes, nursing homes, and hospitals). Categorical variables are presented as numbers and percentages, and inter-group comparisons were conducted using Chi-square and Fisher exact tests. The multivariable Cox regression models identified independent predictors of mortality, including demographic and clinical factors. Kaplan–Meier curves were generated to evaluate the overall survival at one year, and the log-rank test was used to compare survival among different groups. The receiver operating characteristic (ROC) curve analysis was applied to assess diagnostic performance. Threshold values were determined by the Youden index method. A comparison of the AUC curves was performed with a nonparametric approach using the theory of generalized U-statistics to generate an estimated covariance matrix previously reported by DeLong et al. [23]. Significance (*) was accepted at *p* < 0.05 for all statistical analyses.

## 3. Results

### 3.1. Study Population

This study included a total of 236 patients, of whom 59.3% were female and 40.7% were male. The mean age of the participants was 72.6 ± 17.4 years (Supplementary Table S1). The most common indication for PEG placement was neurologic disorders, accounting for 69.9% of the cases, followed by head and neck cancers (11.0%), hemorrhage (8.5%), other cancers (4.7%), and other indications (5.9%). All indications are shown in detail in Supplementary Table S2. Enteral feeding was most frequently administered via pump (89.8%), whereas 10.2% of patients were fed via bolus. In terms of the formula type, 33.5% of patients used hypercaloric formulas, 28.4% diabetic-specific formulas, 25.4% standard formulas, 8.5% immunonutrition formulas, and 4.2% kidney-specific formulas. In terms of the care setting, 54.2% of the patients were followed in hospital, 14.0% in nursing homes, and 31.8% at home. Among those receiving home care, 61.3% had one caregiver, while 38.7% had two or more caregivers. Female caregivers were predominant (80.0%), with a mean caregiver age of 53.4 ± 11.6 years. The educational backgrounds of the caregivers were primarily primary school (68.0%), followed by high school (20.0%), and university education (12.0%). At a median of 6 months following PEG placement, 25.8% of patients required PEG tube replacement. Of these patients, 60.7% underwent one replacement, while 39.3% needed multiple (two or more) replacements. Minor complications were observed in 25.8% of the patients, with the most common being tube dislodgement (14.0%), followed by wound infections (5.9%), tube leakage (4.2%), and tube blockage (1.7%). The 30-day mortality rate was 19.5%, with an overall mortality rate of 32.2%. The median survival time was 38 weeks (95% CI: 35–41 weeks). The demographic and clinical characteristics of the patients are summarized in Supplementary Table S1.

### 3.2. Findings Associated with One-Year Mortality

Table 1 presents the demographic and clinical findings related to one-year mortality following PEG. Patients with PEG indications due to hemorrhage had a higher risk of mortality compared to those with neurological disorders as the reference group (HR: 2.47, 95% CI: 1.28–4.78, *p* = 0.007). An increased CAR level was associated with a higher risk of mortality (HR: 1.1, 95% CI: 1.05–1.16, *p* < 0.001), while a decreased PNI level was linked to an increased mortality risk (HR: 0.91, 95% CI: 0.87–0.94, *p* < 0.001). In terms of feeding methods, pump feeding was linked to a higher mortality risk compared to bolus feeding (HR: 0.10, 95% CI: 0.01–0.69, *p* = 0.020). The use of diabetic-specific formulas showed a higher association with a mortality risk compared to standard formulas (HR: 1.91, 95% CI: 1.01–3.64, *p* = 0.049).

**Figure 1:**
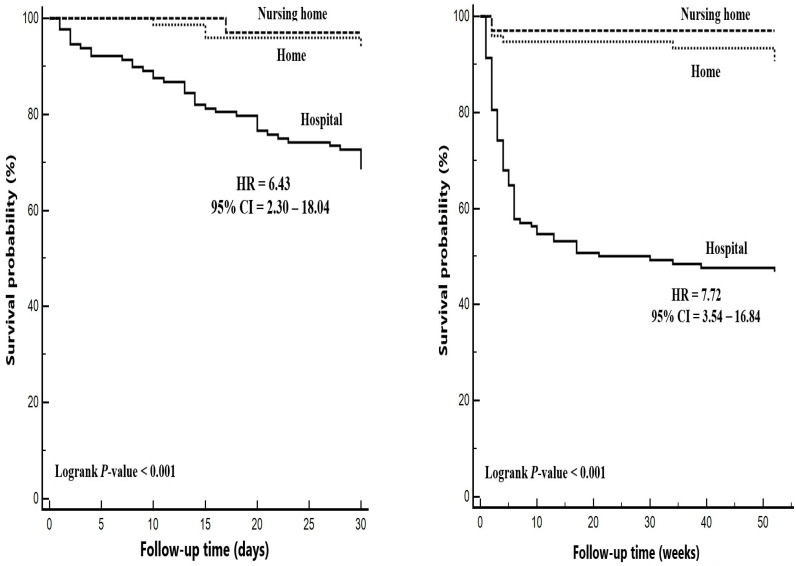
The 30-day (**left**) and one-year (**right**) mortality risks by place of residence.

Patients who were followed in nursing homes (HR: 0.04, 95% CI: 0.01–0.30, *p* = 0.002) or homes (HR: 0.17, 95% CI: 0.08–0.38, *p* < 0.001) had significantly lower mortality risks compared to hospital-based care, and the risk of mortality did not differ significantly between those patients receiving home care and those in nursing homes. However, both the 30-day (HR = 6.43, 95% CI = 2.30–18.04, *p* < 0.001) and one-year mortality risks (HR = 7.72, 95% CI = 3.54–16.84, *p* < 0.001) were higher in hospital-based care settings compared to nursing homes (Figure 1) (Table 2).

PEG tube replacement was associated with a lower mortality risk compared to those who did not require a replacement (HR: 0.12, 95% CI: 0.05–0.34, *p* < 0.001). Additionally, patients who experienced minor complications had a reduced mortality risk (HR: 0.13, 95% CI: 0.05–0.35, *p* < 0.001), with tube dislodgement specifically associated with a lower mortality risk (HR: 0.18, 95% CI: 0.06–0.56, *p* = 0.003) (Table 2).

### 3.3. Independent Predictors of One-Year Mortality

The multivariable regression model (Table 3) was constructed by including the potential risk factors (*p* < 0.25) identified in the univariable regression analysis, as shown in Table 1 and Table 2. A decreased PNI level was independently associated with a higher risk of mortality (HR: 0.93, 95% CI: 0.89–0.97, *p* < 0.001). Patients who were followed in nursing homes (HR: 0.07, 95% CI: 0.01–0.48, *p* = 0.007) and those receiving care at home (HR: 0.20, 95% CI: 0.07–0.54, *p* = 0.002) were independently associated with a lower risk of mortality compared to the patients followed in hospitals. Additionally, the risk of mortality was 5-fold (1/HR) lower in patients who underwent PEG tube replacement compared to those who did not (HR: 0.20, 95% CI: 0.07–0.54, *p* = 0.002) (Table 3).

### 3.4. Diagnostic Performance of PNI and CAR in Predicting One-Year Mortality

**Figure 2:**
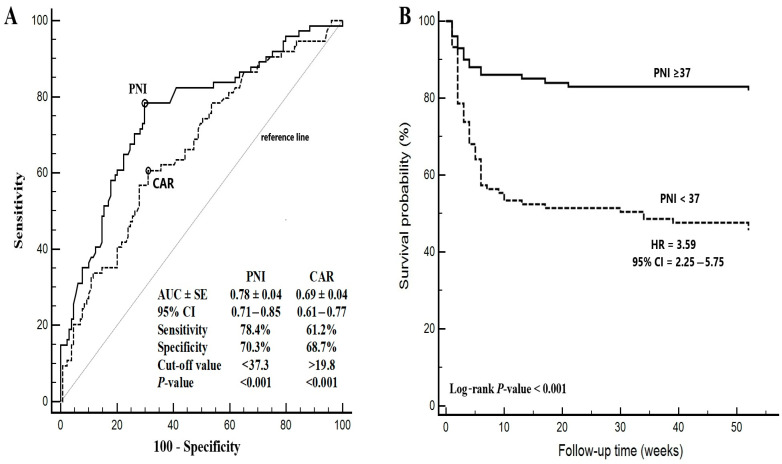
(**A**) The diagnostic performance of the prognostic nutritional index (PNI) and CRP-to-albumin ratio (CAR) in predicting one-year mortality. (**B**) Mortality risk according to the PNI threshold values.

The diagnostic performance of the PNI and the CAR in predicting one-year mortality is presented in Figure 2A. The AUROC for the PNI was higher (0.78 ± 0.04, 95% CI: 0.71–0.85) compared to the CAR (0.69 ± 0.04, 95% CI: 0.61–0.77), indicating that the PNI has better diagnostic accuracy for predicting mortality. The PNI also demonstrated a higher sensitivity (78.4%) and specificity (70.3%) compared to the CAR, which had a sensitivity of 61.2% and specificity of 68.7%. In Figure 2B, the Kaplan–Meier survival curves illustrate the mortality risk according to the PNI threshold value (PNI ≥ 37 vs. PNI < 37). Patients with a PNI < 37 had a 3.59-fold increased risk of mortality compared to those with a PNI ≥ 37 (HR: 3.59, 95% CI: 2.25–5.75, log-rank *p* < 0.001) (Figure 2B).

### 3.5. Demographic and Clinical Findings According to Place of Residence

In patients followed in nursing homes, the rate of females and the mean age were higher than in those followed in hospital or home care settings. Neurological disorders were the most frequent indication in all settings, particularly in nursing home-monitored patients compared to those monitored in hospital and home care settings (93.9% vs. 64.8% vs. 68.0%, *p* < 0.001, respectively). Regarding other PEG indications, the rate of hemorrhage was higher in hospital-monitored patients compared to those monitored in other settings, while head and neck cancers were more prevalent in patients who followed home care. The median CAR level was higher in hospital-monitored patients compared to those monitored in nursing homes and home care settings (17.3 vs. 11.5 vs. 12.0, *p* = 0.019, respectively), while the mean PNI level was lower (37.2 ± 7.9 vs. 40.6 ± 7.1 vs. 40.8 ± 8.8, *p* = 0.040, respectively). Enteral nutrition with a pump was provided to all patients monitored in hospital and nursing home settings, while 68% of patients who were followed in home care used a pump. Patients who were monitored in nursing homes had the highest frequency of PEG tube replacement compared to those receiving home care or hospital settings (57.6% vs. 30.7% vs. 14.8%, *p* < 0.001, respectively). Patients who were followed in nursing homes experienced a higher rate of minor complications compared to those monitored in home care or hospital settings (57.6% vs. 30.7% vs. 14.8%, *p* < 0.001, respectively). Mortality rates were highest among patients receiving hospital-based care compared to other residential settings (Table 4).

## 4. Discussion

To the best of our knowledge, this is the first study in the literature to evaluate the one-year mortality rates and the comparative prognostic value of the PNI and CAR in patients undergoing PEG placement across different care settings. Our findings revealed that care environments significantly influence survival outcomes, with patients monitored at home or in nursing homes demonstrating lower mortality rates compared to those followed in hospitals. Moreover, the PNI outperformed the CAR as a diagnostic tool for predicting one-year mortality, emphasizing its clinical utility in risk stratification. These results underscore the importance of individualized care strategies, proactive complication management, and nutritional optimization in improving the prognosis of PEG patients.

PEG has gained widespread acceptance as a minimally invasive and effective method for long-term enteral feeding in patients who are unable to maintain adequate oral intake due to various etiologies [24,25]. Our results showed that nearly 70% of the PEG indications were related to neurologic disorders, a finding consistent with prior studies indicating that neurologic pathologies are the most frequent reason for PEG placement [1,26,27,28]. Similar to the existing literature, head and neck cancers represented the second most common indication in our cohort, underscoring the importance of establishing adequate nutritional support in patients who develop dysphagia or a high risk of malnutrition due to oncologic treatments [29,30]. PEG placement is followed by a critical period of monitoring that greatly influences the clinical course and complication rates. In hospitals, nursing homes, or home care settings, proper education regarding enteral nutrition, tube care, early identification of complications, and timely intervention are fundamental to determining patient outcomes [5,6]. Home-based care offers certain advantages, including enhanced patient comfort, reduced risk of hospital-acquired infections, and cost-effectiveness. However, for successful care at home, patients or their family members (or caregivers) must possess adequate knowledge and skills regarding tube management and nutritional support [13,31]. Inadequate care may lead to an increased risk of complications such as tube blockage, dislodgement, skin irritation, and local infections [32]. On the other hand, a hospital-based follow-up allows for immediate intervention by specialized personnel, standardization of nutrition protocols, and faster recognition and management of complications. Nonetheless, prolonged hospital stays may lead to an increased risk of nosocomial infections and higher healthcare costs, especially in elderly and comorbid patients [33]. Hence, the decision to continue a follow-up at home or in the hospital should consider the patient’s overall condition, comorbidities, family or caregiver support, and the physical environment at home.

Notably, over half of our patients were monitored in the hospital, whereas about one-third were followed at home, and a smaller proportion was in nursing homes. Interestingly, those who were monitored in nursing homes and at home exhibited lower one-year mortality rates compared to hospital-monitored patients, even after adjusting for potential confounders. Previous studies have reported that the in-hospital mortality rates ranged between 9% and 66% [34,35,36,37,38]. A study comparing nursing home residents and hospitalized patients who underwent PEG revealed that the 30-day and 60-day mortality rates were about six times higher in the hospital group than in the nursing home group [9]. In a study involving patients with PEG placement for various indications, primarily head and neck cancer and dysphagia, it was reported that the 30-day mortality rates were comparable between patients under professional care and those cared for by themselves or their families. However, the one-year mortality rate was higher in the professional care group [10]. Another study involving Parkinson’s patients with PEG placement indicated that the 30-day survival rates did not significantly differ between those followed at home and in nursing homes. In contrast, a long-term follow-up showed better survival outcomes for patients monitored at home [11]. This could be partly explained by the differences in underlying disease severity, selection bias (i.e., patients in better clinical conditions being discharged earlier), and the nature of care in home or nursing home settings, where a multidisciplinary approach and stable environment may mitigate nosocomial complications [39,40].

Our findings are in line with earlier studies that suggest that prolonged hospital stays elevate the risk of hospital-acquired infections and may adversely affect quality of life [33,41]. Interestingly, our results demonstrated that PEG tube replacement was independently associated with a significantly lower mortality risk, with patients undergoing replacement having a 5-fold reduced risk of death compared to those who did not. All PEG tube replacements were performed due to minor complications. More than half of the nursing home-monitored patients required PEG tube replacement, whereas this need was observed in about one-third of the patients followed at home. This finding highlights the potential role of proactive PEG tube management in reducing the mortality risk, likely by preventing complications, such as tube blockage, leakage, or dislodgement, which can lead to inadequate enteral nutrition, infections, or aspiration events. The higher rate of PEG tube replacement in nursing homes and home care settings suggests that the presence of well-trained nursing staff or adequately educated caregivers plays a crucial role in mitigating these risks. Proper tube care, accurate administration of enteral formulas, and early recognition and management of complications could help control the risk of secondary infections, ultimately contributing to a reduction in mortality [12,13]. On the other hand, higher mortality rates in hospital-monitored patients are likely linked to the severity of their underlying conditions, including pronounced nutritional deficiencies, compromised immunity, or an elevated hemorrhage risk.

Several studies have indicated that decreased serum albumin levels, lower lymphocyte counts, and higher CRP levels serve as independent predictors of mortality [9,37,42,43,44,45,46]. However, it has been demonstrated that the PNI and CAR outperform their components for predicting mortality in different diseases [47,48]. Our findings revealed that a pre-replacement PNI was lower, and that the CAR was higher in patients monitored in hospitals compared to those in other care environments, supporting that more critically ill patients with worse nutritional and inflammatory statuses are cared for in hospital settings. To the best of our knowledge, the available studies on these indices in PEG patients are restricted, and none have focused on comparing the diagnostic performance of the two indices. In a study by Adachi et al. involving patients who underwent PEG, 268 patients were retrospectively analyzed to identify the predictors of early mortality [19]. In their multivariable regression model, the PNI was the only prognostic factor identified. The authors determined a PNI cut-off value of 36.7, which demonstrated a sensitivity of 50% and a specificity of 79% for predicting early mortality. In the same study, a prospective analysis was conducted on 115 patients deemed suitable for PEG placement. Patients were grouped based on their PNI values (PNI ≥ 37 and <37). In the better-nutrition group, 79 patients underwent PEG without additional nutritional support, whereas in the poorer-nutrition group, patients received more than 10 days of pre-PEG nutritional support. In the prospective cohort, survival analysis demonstrated no significant difference in 30-day mortality rates between the two groups after PEG placement [19]. In a study conducted by Yoshida et al. involving 100 patients who underwent PEG, low PNI levels were associated with early mortality. However, due to high multicollinearity with controlling nutritional status (CONUT) scores, the PNI was excluded from the multivariable regression analysis [20]. In the present study, the PNI cut-off value and sensitivity for predicting one-year mortality were similar to those reported by Adachi et al. [19]. However, our study demonstrated a higher sensitivity compared to their findings, further supporting the prognostic utility of the PNI in patients who underwent PEG. On the other hand, the sensitivity and specificity values of the CAR aligned with the ranges reported in prior studies (61–91% for sensitivity, 46–79% for specificity) [49,50,51]. Conversely, the PNI demonstrated superior diagnostic performance compared to the CAR in predicting one-year mortality. Collectively, these results highlight the importance of incorporating comprehensive nutritional and inflammatory markers, particularly the PNI, into clinical practice for optimizing prognostication and targeted interventions in PEG patients.

Our results have several clinical implications. First, they underscore the importance of evaluating the immunonutritional status using indices such as the PNI to risk-stratify PEG candidates and implement targeted interventions in those with poor nutritional parameters. Second, they suggest that the place of residence after PEG is not a mere administrative decision but a significant factor influencing patient outcomes, emphasizing the importance of transitional care and caregiver education. Finally, the strong association of timely PEG tube replacement and adequate follow-up with improved survival rates highlights the need for standardized protocols to reduce preventable complications.

### 4.1. Limitations

Our study has several limitations that warrant consideration. First, the retrospective design introduces the potential for selection bias and limits the ability to infer causality. Additionally, this study was conducted at a single center, which could restrict the generalizability of the results to other institutions with different patient populations, care practices, or healthcare systems. Second, while our study considered the impact of key confounding variables, including age, gender, PEG indication, nutritional type, formula type, caregiver characteristics, and minor complications, the effects of unmeasured confounders may have had a significant influence on the study outcomes. Disease severity, frailty indices, functional capacity, and comorbidity burden could have influenced both survival outcomes and the likelihood of hospital-based care. Another consideration is that hospitalized patients inherently represent a more complex and critically ill group, requiring higher levels of medical intervention and multidisciplinary care. This subgroup likely had a greater burden of comorbidities and worse baseline nutritional status, which could explain the higher mortality rate observed in the hospital-monitored patients. Additionally, the decision to hospitalize SGA-positive patients may not have been solely based on their nutritional status but rather influenced by underlying acute conditions requiring inpatient care. These factors introduce potential bias, as patients who remained in non-hospital settings may have been more stable, requiring less intensive care, and thus exhibiting lower mortality rates. Although the study included a detailed assessment of nutritional and inflammatory markers, longitudinal changes in the PNI, CAR, or other clinical variables were not examined during the follow-up period, which could provide a better understanding of how these parameters change over time and impact the clinical outcomes. Third, socioeconomic status and healthcare accessibility may have played a role in determining the post-PEG care settings, potentially biasing the observed mortality differences. Additionally, this study did not prospectively collect or evaluate patient-reported outcomes and quality-of-life measures, which can be crucial for assessing the broader impact of different care settings (hospitals, nursing homes, or homes) on daily functioning and psychosocial well-being. Lastly, while our study presents data on minor complications, it does not include information on major complications such as severe infections, peritonitis, or gastrointestinal perforations, nor does it specify causes of mortality. Including such data could have contributed to a more comprehensive understanding of the clinical outcomes following PEG placement.

### 4.2. Future Directions

To overcome these limitations, future studies should incorporate prospective, multicenter designs with standardized assessments of baseline disease severity, frailty indices, and comorbidity scales to improve risk stratification and minimize selection bias. Additionally, integrating serial measurements of the PNI, CAR, and other inflammatory and nutritional biomarkers over time could provide valuable insights into their prognostic significance and potential for guiding nutritional interventions. Further research should also explore the causative mechanisms behind the observed differences in mortality across care settings. This includes analyzing the impact of healthcare accessibility, caregiver education, and institutional variations in PEG management protocols on patient outcomes. A better understanding of hospital-based mortality patterns—including detailed characterization of underlying pathologies, complication rates, and treatment approaches—could help identify modifiable factors to improve the survival rates in hospitalized patients. Another important area for future research is the assessment of major PEG-related complications, such as peritonitis, severe infections, and gastrointestinal perforations, to provide a more comprehensive picture of PEG outcomes. Additionally, studies incorporating patient-reported outcomes, quality-of-life metrics, and caregiver burden assessments could offer a holistic perspective on the impact of PEG across different care settings. Lastly, the influence of socioeconomic factors, regional healthcare disparities, and healthcare infrastructure on PEG outcomes remains an underexplored area. Future research should aim to identify how these variables affect post-PEG prognosis, access to appropriate care, and long-term patient well-being.

## 5. Conclusions

Our study identified neurological disorders as the most common indication for PEG and demonstrated a significant association between PEG replacement, complications, and one-year mortality rates. Patients managed in nursing homes or at home had lower mortality risks compared to hospitalized patients, highlighting the importance of personalized care settings in facilitating early intervention and reducing nosocomial complications. Additionally, regular PEG tube replacement was associated with a significantly lower risk of mortality, underscoring the need for structured follow-up and proactive tube management to prevent severe tube-related complications. This finding reinforces the importance of well-trained caregivers and standardized care protocols in improving patient outcomes. Furthermore, our study demonstrated that the PNI is a superior prognostic marker compared to the CAR for predicting one-year survival. Given its greater predictive value, integrating the PNI into routine clinical practice may enhance risk stratification, nutritional assessment, and individualized therapeutic planning in PEG patients. From a clinical perspective, these findings support the implementation of a multidisciplinary approach in PEG patient management, incorporating nutritional optimization, proactive tube care, and tailored care environments to minimize complications and improve survival.

## Figures and Tables

**Table 1:** Demographic and clinical findings associated with one-year mortality in post-PEG patients.

Variables	Mortality		Univariable Regression		
	No n = 160	Yes n = 76	HR	95% CI	*p*-Value
Gender, n (%)					
Female	102 (63.8)	38 (50.0)	ref		
Male	58 (36.2)	38 (50.0)	1.55	0.98–2.43	0.056
Age, years	71.8 ± 17.7	74.3 ± 16.8	1.01	0.99–1.02	0.381
Indications, n (%)					
Neurologic disorders	120 (75.0)	45 (59.2)	ref		
Head and neck cancers	19 (11.9)	7 (9.2)	1.04	0.47–2.31	0.920
Other cancer	7 (4.4)	4 (5.3)	1.48	0.53–4.12	0.451
Hemorrhage	9 (5.6)	11 (14.5)	2.47	1.28–4.78	0.007 *
Others	5 (3.1)	9 (11.8)	2.90	1.41–5.94	0.004 *
Laboratory findings					
Albumin	3.2 ± 0.6	2.7 ± 0.5	0.28	0.18–0.44	<0.001 *
Lymphocytes	1.8 (1.1–2.5)	1.3 (0.9–2.0)	0.7	0.53–0.91	0.008 *
CRP	34.4 (11.9–75.4)	57.4 (26.8–107.1)	1.03	1.01–1.05	0.010 *
CAR	11.1 (4.1–24.7)	22.3 (10.0–46.5)	1.1	1.05–1.16	<0.001 *
PNI	41.4 ± 8.0	34.3 ± 7.3	0.91	0.87–0.94	<0.001 *
Feeding method, n (%)					
Pump	137 (85.6)	75 (98.7)	ref		
Bolus	23 (14.4)	1 (1.3)	0.10	0.01–0.69	0.020 *
Formula type					
Standard	46 (28.7)	14 (18.4)	ref		
Hypercaloric	54 (33.8)	25 (32.9)	1.43	0.74–2.74	0.288
Diabetic	40 (25.0)	27 (35.5)	1.91	1.01–3.64	0.049 *
Immunonutrition	15 (9.4)	5 (6.6)	1.09	0.39–3.03	0.869
Kidney specific	5 (3.1)	5 (6.6)	2.33	0.84–6.47	0.105

Data are mean ± standard deviation or median (IQR) or number (%). * *p*-value < 0.05 shows statistical significance. Abbreviations: CI, confidence interval; CRP, *C*-reactive protein; CAR, CRP-to-albumin ratio; HR, hazard ratio; PNI, prognostic nutritional index.

**Table 2:** The effects of living environment, caregiver characteristics, and complications on one-year mortality in post-PEG patients.

Variables	Mortality		Univariable Regression		
	No n = 160	Yes n = 76	HR	95% CI	*p*-Value
Place of residence, n (%)					
Hospital	60 (37.5)	68 (89.5)	ref		
Nursing home	32 (20.0)	1 (1.3)	0.04	0.01–0.30	0.002 *
Home	68 (42.5)	7 (9.2)	0.17	0.08–0.38	<0.001 *
Characteristics of home caregivers					
Number of caregivers, n (%)					
One	44 (64.7)	2 (28.6)	ref		
Two or more	24 (35.3)	5 (71.4)	4.31	0.84–22.23	0.281
Gender, n (%)					
Female	55 (80.9)	5 (71.4)	ref		
Male	13 (19.1)	2 (28.6)	1.56	0.30–8.07	0.592
Age, years	53.4 ± 10.9	54.0 ± 18.3	1.01	0.94–1.08	0.921
Education, n (%)					
University	8 (11.8)	1 (14.3)	ref		
High school	14 (20.6)	1 (14.3)	0.56	0.04–8.97	0.683
Primary school	46 (67.6)	5 (71.4)	0.86	0.10–7.38	0.892
PEG tube replacement, n (%)					
No	103 (64.4)	72 (94.7)	ref		
Yes	57 (35.6)	4 (5.3)	0.12	0.05–0.34	<0.001 *
Number of replacements, n (%)					
One	33 (20.6)	4 (5.3)	ref		
Two or more	24 (15.0)	-	0.02	0.01–82.89	0.362
Minor complications, n (%)					
No	103 (64.4)	72 (94.7)	ref		
Yes	57 (35.6)	4 (5.3)	0.13	0.05–0.35	<0.001 *
Wound infection	14 (8.8)	-	0.01	0.01–69.6	0.996
Tube leakage	10 (6.2)	-	0.00	0.01–21.83	0.971
Tube dislodgement	30 (18.8)	3 (3.9)	0.18	0.06–0.56	0.003 *
Tube blockage	3 (1.9)	1 (1.3)	0.48	0.07–3.47	0.470

Data are mean ± standard deviation or median (IQR) or number (%). * *p*-value < 0.05 shows statistical significance. Abbreviations: CI, confidence interval; HR, hazard ratio; PEG, percutaneous endoscopic gastrostomy.

**Table 3:** Independent predictors associated with one-year mortality.

Variables	Multivariable Regression		
	HR	95% CI	*p*-Value
PNI	0.93	0.89–0.97	<0.001 *
Place of residence			
Hospital	ref		
Nursing home	0.07	0.01–0.48	0.007 *
Home	0.20	0.07–0.54	0.002 *
PEG tube replacement	0.20	0.07–0.54	0.002 *
	−2Log Likelihood = 669.2		

The multivariable regression model incorporated the potential risk factors (*p* < 0.25) listed in Table 1 and Table 2. * *p*-value < 0.05 shows statistical significance. Abbreviations: CI, confidence interval; HR, hazard ratio; PEG, percutaneous endoscopic gastrostomy; PNI, prognostic nutritional index.

**Table 4:** Distribution of demographic and clinical findings according to the place of residence of patients after PEG.

Variables	Place of Residence			*p*-Value
	Hospital	Nursing Home	Home	
	n = 128	n = 33	n = 75	
Gender, n (%)				
Female	71 (55.5)	26 (78.8)	43 (57.3)	0.048 *
Male	57 (44.5)	7 (21.2)	32 (42.7)	
Age, years	72.5 ± 16.6	**79.6 ± 13.6**	69.8 ± 19.5	0.027 *
Indications, n (%)				
Neurologic disorders	83 (64.8)	31 (93.9)	51 (68.0)	<0.001 *
Head and neck cancers	9 (7.0)	1 (3.0)	16 (21.3)	
Other cancer	7 (5.5)	1 (3.0)	3 (4.0)	
Hemorrhage	17 (13.3)	-	3 (4.0)	
Others	12 (9.4)	-	2 (2.7)	
Laboratory findings				
Albumin	**2.9 ± 0.5**	3.1 ± 0.5	3.2 ± 0.7	0.003 *
Lymphocytes	1.7 (1.0–2.2)	1.7 (0.9–2.6)	1.7 (0.9–2.2)	0.947
CRP	47.8 (22.4–97.8)	33.2 (8.4–93.0)	35.2 (11.9–67.4)	0.055
CAR	**17.3 (7.6–35.6)**	11.5 (3.1–24.1)	12.0 (4.2–20.1)	0.019 *
PNI	**37.2 ± 7.9**	40.6 ± 7.1	40.8 ± 8.8	0.040 *
Feeding method, n (%)				
Pump	128 (100.0)	33 (100.0)	51 (68.0)	<0.001 *
Bolus	-	-	24 (32.0)	
Formula type				
Standard	25 (19.5)	13 (39.4)	22 (29.3)	0.130
Hypercaloric	47 (36.7)	7 (21.2)	25 (33.3)	
Diabetic	42 (32.8)	8 (24.2)	17 (22.7)	
Immunonutrition	9 (7.0)	2 (6.1)	9 (12.0)	
Kidney specific	5 (3.9)	3 (9.1)	2 (2.7)	
PEG tube replacement, n (%)				
No	109 (85.2)	14 (42.4)	52 (69.3)	<0.001 *
Yes	19 (14.8)	19 (57.6)	23 (30.7)	
Number of replacements, n (%)				
One	16 (84.2)	9 (47.4)	12 (52.2)	0.041 *
Two or more	3 (15.8)	10 (52.6)	11 (47.8)	
Minor complications, n (%)				
No	109 (85.2)	14 (42.4)	52 (69.3)	<0.001 *
Yes	19 (14.8)	19 (57.6)	23 (30.7)	
Wound infection	3 (2.3)	7 (21.2)	4 (5.3)	<0.001 *
Tube leakage	3 (2.3)	2 (6.1)	5 (6.7)	
Tube dislodgement	11 (8.6)	10 (30.3)	12 (16.0)	
Tube blockage	2 (1.6)	-	2 (2.7)	
30-day mortality, n (%)	41 (32.0)	1 (3.0)	4 (5.3)	<0.001 *
One-year mortality, n (%)	68 (53.1)	1 (3.0)	7 (9.3)	<0.001 *

Data are mean ± standard deviation or median (IQR) or number (%). * *p*-value < 0.05 shows statistical significance. Bold characters show the difference between groups. Abbreviations: CRP, *C*-reactive protein; CAR, CRP-to-albumin ratio; PEG, percutaneous endoscopic gastrostomy; PNI, prognostic nutritional index.

## Data Availability

The data that support the findings of this study are available on request from the corresponding author due to privacy and ethical reasons.

## Supplementary Materials

The following supporting information can be downloaded at: https://www.mdpi.com/article/10.3390/nu17050904/s1, Table S1: Demographic and clinical findings of study population; Table S2: Indication for insertion of a percutaneous endoscopic gastrostomy tube.
