# Supplementary material for: One-Year Mortality After Percutaneous Endoscopic Gastrostomy: The Prognostic Role of Nutritional Biomarkers and Care Settings
Source: Nutrients. 2025 Mar 5;17(5):904. doi: 10.3390/nu17050904 (PMC11901879; doi:10.3390/nu17050904)
Supplement: Supplementary file 1 [file nutrients-17-00904-s001.zip › nutrients-3491873-supplementary.pdf]

Supplement Table S1. Demographic and clinical findings of study population.

Variables	All population n = 236
Gender, n (%)	
Female	140 (59.3)
Male	96 (40.7)
Age, years	72.6 ± 17.4
Indications, n (%)	
Neurologic disorders	165 (69.9)
Head and neck cancers	26 (11.0)
Other cancer	11 (4.7)
Hemorrhage	20 (8.5)
Others	14 (5.9)
Laboratory findings	
Albumin, g/dL	3.0 ± 0.6
Lymphocytes, $\times 10^3/\mu L$	1.7 (1.0-2.2)
CRP, mg/L	43.6 (17.4-83.9)
CAR	14.6 (5.2-32.6)
PNI	38.8 ± 8.5
Feeding method, n (%)	
Pump	212 (89.8)
Bolus	24 (10.2)
Formula type	
Standard	60 (25.4)
Hypercaloric	79 (33.5)
Diabetic	67 (28.4)
Immunonutrition	20 (8.5)
Kidney specific	10 (4.2)
Place of residence, n (%)	
Hospital	128 (54.2)
Nursing home	33 (14.0)
Home	75 (31.8)
Characteristics of home caregivers	
Number of caregivers, n (%)	
One	46 (61.3)
Two or more	29 (38.7)
Gender, n (%)	
Female	60 (80.0)
Male	15 (20.0)
Age, years	53.4 ± 11.6
Education, n (%)	
University	9 (12.0)
High school	15 (20.0)
Primary school	51 (68.0)
PEG tube replacement, n (%)	
No	175 (74.2)
Yes	61 (25.8)
Number of replacement, n (%)	
One	37 (60.7)
Two or more	24 (39.3)
Minor complications, n (%)	
No	175 (74.2)
Wound infection	14 (5.9)
Tube leakage	10 (4.2)
Tube dislodgement	33 (14.0)
Tube blockage	4 (1.7)

One-year mortality, n (%)	76 (32.2)
Median survival time, weeks (95% CI)	38 (35-41)

Data are mean ± standard deviation or median (IQR), or number. Abbreviations: CRP, C-reactive protein; CAR, CRP to albumin ratio; PEG, percutaneous endoscopic gastrostomy; PNI, prognostic nutritional index.

Supplement Table S2. Indication for insertion of a percutaneous endoscopic gastrostomy tube.

Indications	n	All population %	Sub-diagnosis %
Neurologic disorders			
Alzheimer	62	26.3	37.6
Cerebral palsy	2	0.8	1.2
Cerebrovascular event	64	27.1	38.8
Dementia	12	5.1	7.3
Hydrocephalus	1	0.4	0.6
Hypoxic encephalopathy	2	0.8	1.2
Motor neurone disease	3	1.3	1.8
Parkinson	5	2.1	3.0
Psychomotor retardation	1	0.4	0.6
Trauma	12	5.1	7.3
Uremic encephalopathy	1	0.4	0.6
Hemorrhage	20	8.5	100.0
Head and neck cancers			
Brain	11	4.7	42.3
Esophagus	3	1.3	11.5
Larynx	5	2.1	19.2
Pharynx	1	0.4	3.8
Salivary gland	1	0.4	3.8
Thyroid	1	0.4	3.8
Tongue	3	1.3	11.5
Tonsil	1	0.4	3.8
Other cancer			
Breast	3	1.3	27.3
Lung	5	2.1	45.5
Stomach	3	1.3	27.3
Others			
Cardiovascular disease	8	3.4	57.1
Hip fracture or arthroplasty	3	1.3	21.4
Others	3	1.3	21.4
